# A large family with inherited optic disc anomalies: a correlation between a new genetic locus and complex ocular phenotypes

**DOI:** 10.1038/s41598-017-07730-7

**Published:** 2017-08-10

**Authors:** Decai Wang, Xinyuan Pan, Jiangdong Ji, Shun Gu, Xiantao Sun, Chao Jiang, Weiyi Xia, Zhihua Qiu, Xiaoli Kang, Sijia Ding, Qinghuai Liu, Xue Chen, Fang Lu, Chen Zhao

**Affiliations:** 1 Department of Preventive Ophthalmology, Zhongshan Ophthalmic Center, Sun Yat-sen University, Guangzhou, 510060 China; 2 Department of Ophthalmology, The Affliated Wuhu No. 2 People’s Hospital of Wannan Medical College, Wuhu, 241000 China; 3 Department of Ophthalmology, The First Affiliated Hospital of Nanjing Medical University and State Key Laboratory of Reproductive Medicine, Nanjing Medical University, Nanjing, 210029 China; 4 Department of Ophthalmology, Wuxi People’s Hospital affiliated to Nanjing Medical University, Wuxi, 214023 China; 5 Department of Ophthalmology, Children Hospital of Zhengzhou, Zhengzhou, 450053 China; 6 Department of Ophthalmology, Dongyuan Hospital of traditional Chinese medicine, Heyuan, 517000 China; 7 Department of Ophthalmology, Xinhua Hospital, Shanghai Jiao Tong University School of Medicine, Shanghai, 200092 China; 8 Department of Ophthalmology and Vision Science, Eye & ENT Hospital, Shanghai Medical College, Fudan University, Shanghai, 200031 China; 9 Key Laboratory of Myopia of State Health Ministry (Fudan University) and Shanghai Key Laboratory of Visual Impairment and Restoration, Shanghai, 200031 China; 10 Department of Ophthalmology, West China Hospital, Sichuan University, Chengdu, 610041 China

## Abstract

Congenital cavitary optic disc anomalies (CODA) is clinically typified by an enlarged excavation of optic disc in diverse degrees. Here, we report the clinical and genetic findings in a four-generation Chinese family with a complicated form of autosomal dominant CODA. Cardinal manifestations included bilateral excavated optic disc with multiple cilioretinal vessels emerging and bilateral retinoschisis with great variability in the range of extension and severity. Other intra-familial phenotypic diversities were also noted, including severity in retinal atrophy, onset age of visual impairment and presence of congenital nystagmus and strabismus. Genome-wide linkage analysis and fine mapping mapped a novel locus for CODA to a 34.3 cM interval between D14S972 and D14S139 at 14q12-q22.1. A maximum multi-point log odds score of 3.901 was reached at D14S275. However, no mutation was identified by exome sequencing or direct sequencing of *PAX6* and *PAX2* genes, suggesting that the mutation may reside within a regulatory element. In conclusion, we find retinoschisis as a necessary consequence of optic nerve head (ONH) anomalies. The complicated phenotype observed in the family provided additional insights into the inherited ONH anomalies. Mapping of a novel locus, 14q12-q22.1, implies a new disease-causing gene and potential distinct pathogenesis for CODA.

## Introduction

Congenital cavitary optic disc anomalies (CODA) are a group of clinically heterogeneous diseases including optic pit, optic nerve head (ONH) coloboma, morning glory disk anomaly, peripapillary staphyloma, and vacant optic disc^1^. All these malformations are characterized by an enlarged excavation of optic disc in different degrees causing mild or severe visual impairments and even strabismus since childhood^2^. Most CODA cases are sporadic and unilateral. Multigenerational families inherited with optic disc anomalies were rare^1, 3, 4^. Mutations in paired box protein Pax-6 (*PAX6*; MIM: 607108) gene have been reported as disease causative for diverse ONH anomalies, including coloboma, morning glory disc anomaly, optic-nerve hypoplasia/aplasia, and persistent hyperplastic primary vitreous^5^. Mutations in *PAX2* (MIM: 167409) gene are implicated in Papillorenal syndrome (also termed as renal coloboma syndrome) presenting both renal and optic disc anomalies^6^. Commonly observed manifestations of the kidney in Papillorenal syndrome are renal hypoplasia and vesicoureteric reflux leading to end-stage renal disease. The 12q locus, which was later identified as a triplication upstream of the matrix metalloproteinase 19 (*MMP19*, MIM: 601807) gene, was the only assigned genetic locus for CODA^7, 8^.

Here, we reported a four-generation family with autosomal dominant CODA. All affected members not only presented ONH defects, but also showed various grades of retinoschisis, retinal pigment epithelium (RPE)/choroidal atrophy, decreases in best corrected visual acuity (BCVA), and visual field (VF) defects. Some younger patients in this family appeared to have more severe phenotypes showing early onset ages of visual impairment accompanied with congenital nystagmus and strabismus. Using genome-wide linkage analysis, a novel disease locus was assigned to the long arm of chromosome 14 in this family and a maximum multi-point log odds (LOD) score of 3.901 was reached at marker D14S275.

## Results

### Clinical findings

**Figure 1:**
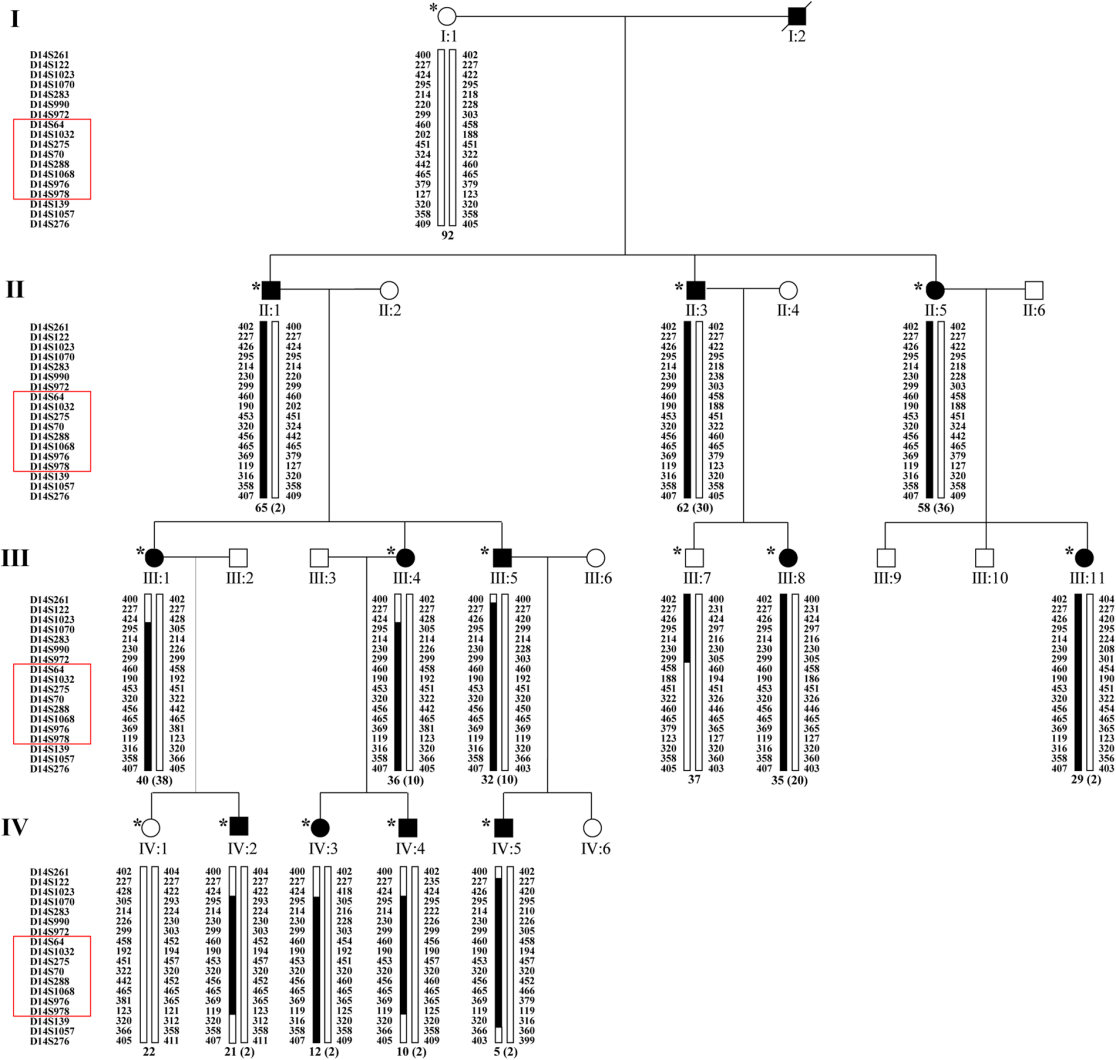
Pedigree of family CL and haplotype reconstruction for the mapped region on chromosome 14. Filled and open symbols represent affected and unaffected members, respectively. The proband is indicated by the black arrow. Haplotypes for tested microsatellite markers in the mapped region and those flanking it are given for all participants. Black bars represent the ancestral haplotype associated with the disease. *Individuals from whom blood samples were collected. The mapped region flanked by markers D14S972 and D14S139 was shared by all patients and was absent in all unaffected members.

**Figure 2:**
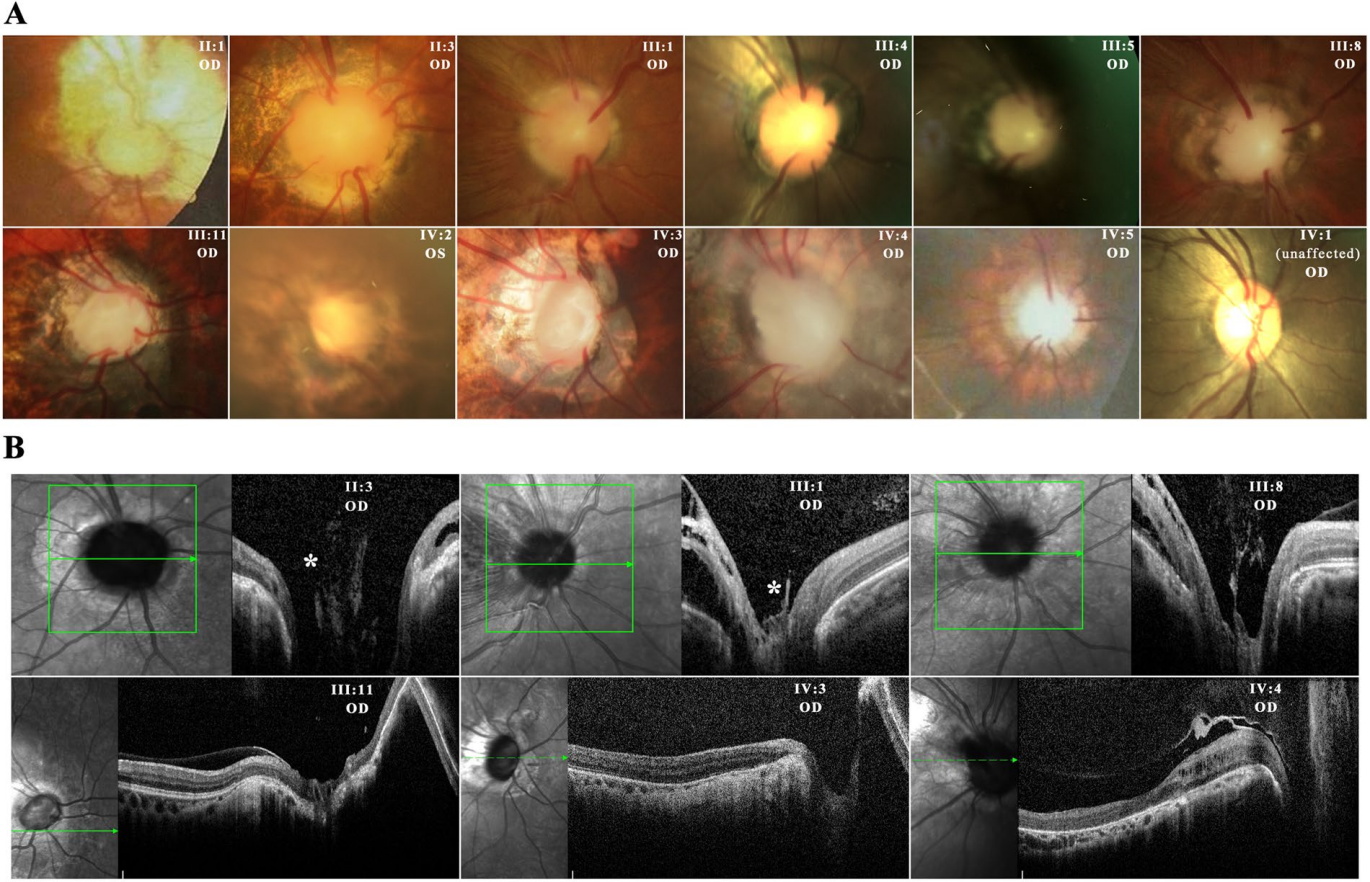
Fundus photos and SD-OCT images suggest ONH anomalies in patients from family CL. (**A**) Fundus photos of 11 affected individuals (II:1, II:3, III:1, III:4, III:5, III:8, III:11, IV:2, IV:3, IV:4, and IV:5) revealed pale-appeared ONH, enlarged excavation with poor defined optic disc rim, and several cilioretinal vessels emanated from the edge of the vacant optic disc. No central retinal artery/vein trunk was observed. ONH of the unaffected member IV:1 was shown as control. OD, right eye; OS, left eye. (**B**) SD-OCT of six affected members (II:3, III:1, III:8, III:11, IV:3, and IV:4) showed steep cupping with glial tissue (indicated by asterisk). Green arrowed lines denote scanned axis by SD-OCT.

Family CL is a four-generation large Chinese family with autosomal dominant CODA (Fig. 1). Twelve affected individuals and 3 unaffected members were recruited with their clinical details summarized in Table 1. All included patients had normal intraocular pressure (IOP) and unremarkable findings in anterior segment, while dilated fundus examination revealed ONH malformation in both eyes of all 12 patients. The abnormal ONHs in these patients were normal or smaller in size with enlarged excavation and poorly defined optic disc rim, resulting in pale-appeared ONHs. The most consistent phenotype among all patients was the abnormally structured vasculatures on ONHs. Several cilioretinal vessels emanated from the edge of the vacant optic disc with no central retinal artery/vein trunk observed (Fig. 2A). Spectral domain-optical coherence tomography (SD-OCT) showed a steep cupping and glial tissue overlying the optic disc in all patients examined (Fig. 2B). No clear break of neural retina and RPE was detected at the blurred edge of ONH in these eyes (Fig. 2B).

**Table 1:** Clinical features of patients from family CL.

ID	Onset Ages (years)*	Age (years)/Sex	BCVA		Exotropia/Nystagmus	Fundus Appearance					Retinoschisis†		VF	
			O.D.	O.S.		ODC	CV	PTA	PC	CA	O.D.	O.S.	O.D.	O.S.
II:1	2	65/M	0.15	HM/10 cm	Yes/Yes	Pale	Yes	Yes	No	Yes	Yes	Yes	NA	NA
II:3	30	62/M	0.8	0.125	Yes/No	Pale	Yes	Yes	NA	Yes	Stage 1	Stage 3	EBS	DL
II:5	36	58/F	0.15	0.25	No/No	Pale	Yes	Yes	Yes	Yes	Yes	Yes	NA	NA
III:1	38	40/F	0.4	0.05	No/No	Pale	Yes	Yes	No	Yes	Stage 3	Stage 3	EBS	DL
III:4	10	36/F	NA	NA	No/No	Pale	Yes	Yes	Yes	No	Yes	Yes	NA	NA
III:5	10	32/M	0.05	1.0	No/No	Pale	Yes	Yes	Yes	No	Yes	Yes	NA	NA
III:8	20	35/F	0.6	0.5	No/No	Pale	Yes	Yes	No	No	Stage 2	Stage 1	EBS	EBS
III:11	2	29/F	0.04	0.25	Yes/Yes	Pale	Yes	Yes	No	Yes	Stage 1	Stage 3	DL	EBS
IV:2	2	21/M	0.2	0.1	Yes/Yes	Pale	Yes	Yes	No	Yes	Yes	Yes	NA	NA
IV:3	2	12/F	0.05	0.15	Yes/Yes	Pale	Yes	Yes	No	Yes	Stage 1	Stage 2	EBS	normal
IV:4	2	10/M	0.1	1.0	No/Yes	Pale	Yes	Yes	No	Yes	Stage 1	Stage 2	NA	NA
IV:5	2	5/M	0.8	0.02	Yes/Yes	Pale	Yes	Yes	No	Yes	Yes	Yes	NA	NA

Abbreviations: F: female; M: male; BCVA: best corrected visual acuity; O.D.: right eye; O.S.: left eye; HM: hand moving; NA: not available; ODC: optic disk color; CV: cilioretinal vessels; PTA: peripapillay textural anomaly; PC: pigmental change; CA: choroidal atrophy; VF: visual field; EBS: enlarged blind spot; DL: difussed loss.

*Onset ages of visiual impairment; †stages of retinoschisis was determined based on SD-OCT images.

**Figure 3:**
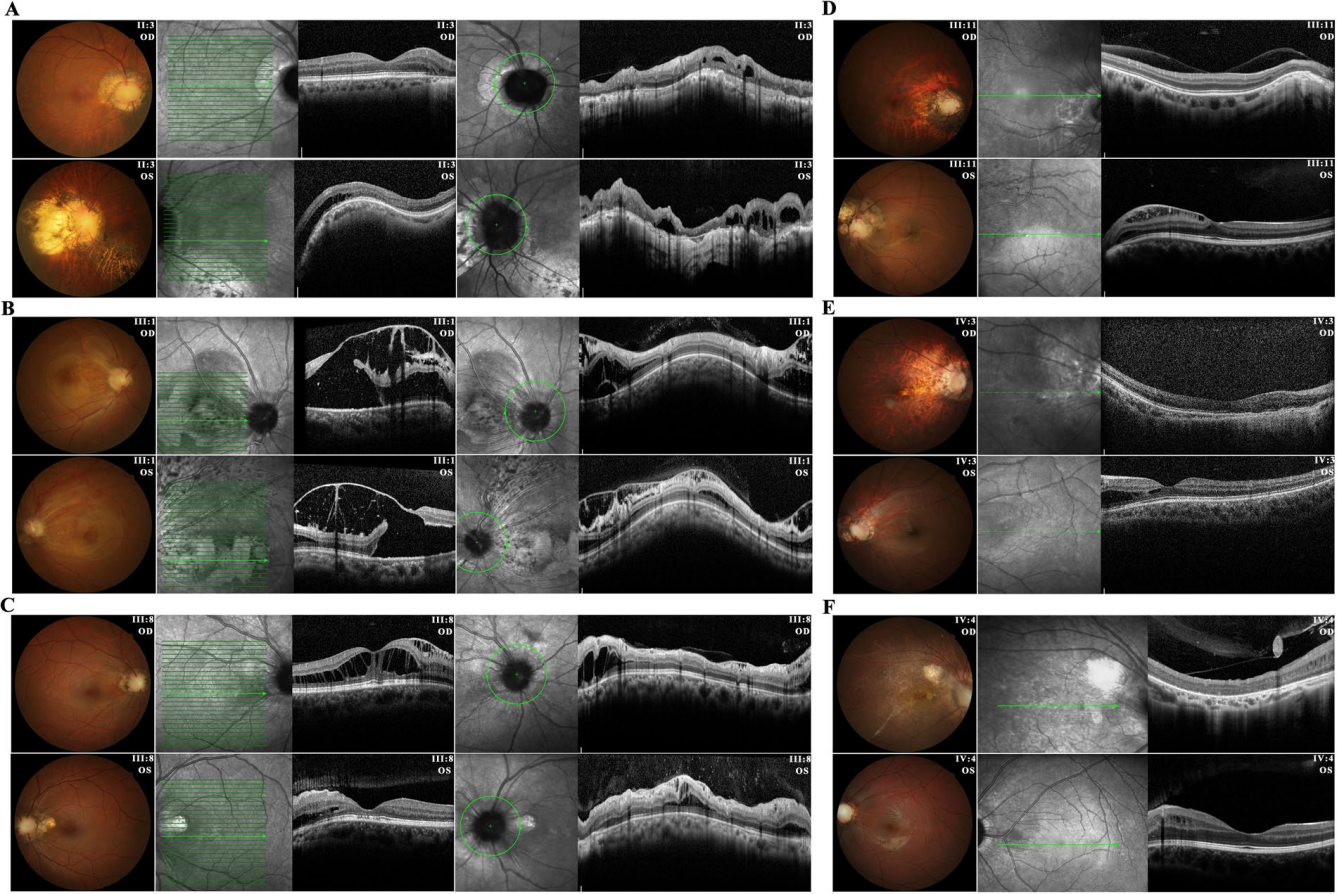
Fundus photos and SD-OCT images show retinoschisis and retinal/choroidal atrophy in six patients from family CL. (**A**) Fundus of patient II:3 presented retinal artery distortion, irregular RPE depigmentation and choroidal atrophy in both eyes. SD-OCT showed peripapillary retinoschisis in right eye, and retinoschisis extended to maculae and inferior temporal quadrant in left eye. (**B**) Fundus photo showed retinoschisis in both eyes of patient III:1 extended beyond the posterior pole. SD-OCT revealed bilateral peripapillary retinoschisis and cystic degeneration in the macular region with vitreomacular traction. (**C**) SD-OCT of patient III:8 revealed bilateral peripapillary retinoschisis and typical retinoschisis between inner nuclear layer (INL) and outer nuclear layer (ONL) with fovea involved in right eye, and close to fovea in left eye. (**D**) Funduscopy of patient III:11, who presented nystagmus, showed peripapillary retinoschisis, retinal artery distortion, and leopard-like choroidal atrophy in both eyes, and retinoschisis extended beyond posterior pole in left eye. SD-OCT showed epi-macular membrane in right eye, and typical retinoschisis within ganglial cells involving fovea in left eye. **(E)** The fundus of patient IV:3 demonstrated coloboma surrounding abnormal ONH and diffused leopard-like choroidal atrophy in both eyes. SD-OCT showed maculoschisis between INL and ONL in left eye and bilateral retinal atrophy. (**F**) Fundus photos and SD-OCT images of patient IV:4 revealed severe retinal degeneration with epi-macular membrane and vitreomacular traction in right eye, and typical retinoschisis within INL in left eye. Green arrowed lines denote scanned axis by SD-OCT. OD, right eye; OS, left eye.

Apparent retinoschisis was detected in all affected members by fundus examination and fundus photography (FP), but varied greatly intra- and inter-individually in both extension and severity (Table 1). Six patients, including II:3, III:1, III:8, III:11, IV:3 and IV:4, received delicate SD-OCT scanning. Based on SD-OCT images, retinoschisis in the 12 eyes of the 6 patients were further categorized into 3 stages by the area involved as stage 1: peripapillary retinoschisis (5 eyes); stage 2: peripapillary retinoschisis with fovea involvement (3 eyes); stage 3: retinoschisis extended beyond the posterior pole (4 eyes) (Fig. 3, Table 1). SD-OCT also revealed different patterns of retinoschisis, including typical schisis within neuron layers (ganglial cell layer, inner nuclear layer and outer nuclear layer), neuron layers cystic degeneration, retinoschisis with vitreomacular traction, and epi-macular membrane on the vitreoretinal surface (Fig. 3). RPE and choroidal atrophies were observed in all patients but varied on locations, neither paralleled to age nor the severity of retinoschisis (Table 1).

VF tests were performed in 5 patients, including II:2, III:1, III:8, III:11 and IV:3. Enlarged physiologic blind spots were revealed in 6 of the 10 eyes tested. Three eyes presented diffused VF defects correlated with expanded retinal/RPE atrophy or retinoschisis showed on SD-OCT (Table 1). For instance, OCT showed peripapillary retinoschisis in both eyes of patient II:3 with extension to inferior temporal quadrant on his left eye (Fig. 3A), which correlated to a superior temporal VF defect (Figure S1).

The 12 affected individuals suffered from visual impairment including metamorphopsy and progressive decrease in vision, but with significantly varied onset ages ranging from early childhood to mid-life (Table 1). Since medical records were not obtainable from most patients in family CL, their onset ages of visual impairments were therefore determined based on careful inquiries on their disease causes, which might influence the accurate timing of onset of vision loss a bit. Twenty of 22 eyes tested had decreased BCVA, ranging from 0.02 to 0.8 (Table 1). Notably, a trend of more severe symptoms toward younger patients in family CL was noted. All patients from the fourth generation and the youngest patient from the third generation (III:11) had very early onset ages of visual impairment and severe congenital nystagmus and strabismus. The proband III:8 experienced metamorphopsy and vision loss since age 20, but still had relatively preserved BCVA at her last visit (Table 1). OCT revealed retinoschisis and RPE atrophy around her center excavated optic disc (Fig. 3C). Compared to III:8, patients III:11 and IV:3 suffered from severe visual impairments since early childhood, and presented congenital exotropia and nystagmus (Table 1). Consistently, their fundus presentations were dramatically changed showing diffused coloboma surrounding abnormal ONH, retinal artery distortion and leopard-like choroidal atrophy (Fig. 3D,E). Patient IV:4 had more severe visual impairment and retinal atrophy in his right eye than left eye (Fig. 3F and Table 1). Interestingly, all three young patients (III:11, IV:3 and IV:4) presented only stage 1 retinoschisis in their right eyes but with significant decreases in BCVA, suggesting a disassociation between visual impairment with retinoschisis.

All patients in family CL denied symptoms or history of renal diseases. Urinary B-scan and renal function tests revealed no remarkable findings on three patients, including II:1 (aged 65), II:3 (aged 62) and III:1 (aged 40).

### Mutation screening in PAX2 and PAX6

We screened for mutations in family CL in two genes, *PAX2* and *PAX6*, both of which were previously implicated in optic disc abnormalities. Promoter region and exons with flanking intronic sequences of the 2 genes were screened using Sanger sequencing on affected individuals III:8 and IV:3. No pathogenic variant was detected.

### Genome-wide linkage analysis and haplotype construction

**Figure 4:**
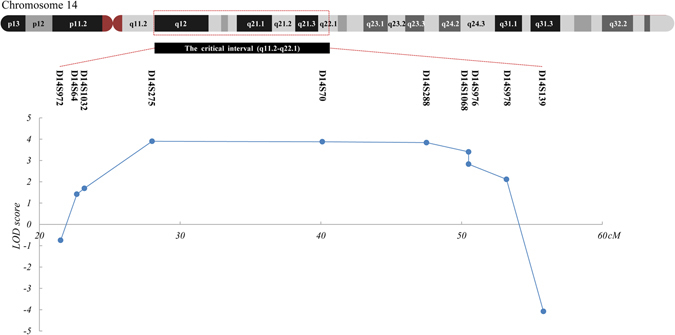
Assignment of the locus for CACD to chromosomal region 14q12-q22.1. Relative order of genotyped microsatellite markers are shown at the bottom next to an ideogram of chromosome 14. Results from multipoint linkage analysis and genetic locations for the markers genotyped are shown below.

Genome-wide linkage analysis was performed using 366 microsatellite markers representing all chromosomes with ~10 centi Morgan (cM) interval. Significant exclusion of linkage was found at all markers with exception of 16 markers, among which, 9 markers on chromosome 14 supported linkage with the disease (Supplementary Table 1). The maximum multipoint LOD score of 3.84 was obtained at D14S288 (penetrance considered as 99%). Subsequently, 12 additional microsatellite markers between D14S261 and D14S176 were selected and genotyped to refine the critical interval linked to CODA in family CL (Fig. 1). Close linkage without recombination was found at markers D14S1070, D14S283, D14S990, D14S972, D14S64, D14S1032, D14S275, D14S70, D14S288, D14S1068, D14S976 and D14S978. Significant positive multi-point LOD scores of 1.676–3.901 were obtained at 8 continuous microsatellite markers including D14S64, D14S1032, D14S275, D14S70, D14S288, D14S1068, D14S976 and D14S978 (Fig. 4 and Table 2). A common haplotype flanked by the D14S1023 and D14S139 was shared by all affected members (Fig. 1). Further, a centromeric recombination between D14S972 and D14S64 was observed on an unaffected member III:7 (Fig. 1), who was 37 of age and free of oculopathy. This finding further refined the disease associated interval to a 34.3 cM (~31 mega base pairs; Mbp) region between D14S972 and D14S139 on chromosomal 14q12-q22.1 (Fig. 4). The exact nucleotide position of the critical region mapped by microsatellite genotyping is 14:24347942–53502746. A total of 17 microsatellite markers on chromosome 12, including markers flanking the *MMP19* gene (D12S85 and D12S83), were examined. Based on our results, the disease causing mutation for this family was not likely located on the upstream of the *MMP19* gene or the 12q locus (Supplementary Table 2).

**Table 2:** Log odds scores of microsatellite markers in chromosome 14.

Chr	Markers	Genetic marshfield (cM)	LOD scores (penetrance 99%)
14	D14S261	6.46	−3.907
14	D14S122	9.36	−3.97
14	D14S1023	8.28	−4.056
14	D14S1070	9.36	−4.076
14	D14S283	13.89	−4.096
14	D14S990	14.6	−1.345
14	D14S972	21.51	−0.743
14	D14S64	22.66	1.416
14	D14S1032	23.2	1.693
14	D14S275	28.01	3.901
14	D14S70	40.11	3.879
14	D14S288	47.51	3.839
14	D14S1068	50.5	3.403
14	D14S976	50.5	2.828
14	D14S978	53.19	2.117
14	D14S139	55.82	−4.08
14	D14S1057	55.82	−4.064
14	D14S276	56.36	−4.086

Abbreviations: Chr, chromosome; cM, centi Morgan.

### Exome Sequencing

To identify the causative mutation for family CL, whole exome sequencing (WES) was performed on 2 affected individuals including III:8 and IV:3. A total of 102 single nucleotide variants and 17insertion/deletions (Indels) were initially identified in both patients. Among them 97 single nucleotide variations (SNVs), including non-coding variants and SNVs found in 5 single nucleotide polymorphism (SNP) databases, were then removed. Co-segregation analysis with Sanger sequencing further revealed that none of the 22 remaining variants cosegregated with the disease phenotype in family CL. In addition, none of the 22 SNVs located within the mapped region. We next tried to use data from exome sequencing to revise the interval. Seven variants located within the interval were heterozygously shared by both screened cases (Supplementary Table 3). Sanger sequencing was then conducted on all 7 variants for co-segregation analysis among all included family members. All 7 variants co-segregated with the disease phenotype for this family, suggesting linkage with the disease. Thus, based on current data from exome sequencing, we were not able to revise the interval.

## Discussion

In this study, we described the clinical presentations in a four-generation family with autosomal dominant CODA, including 100% presence of retinoschisis, and additional variable ocular conditions. By means of genome-wide linkage analysis and fine mapping, we mapped a novel locus for CACD in this family to a 34.3 cM interval between D14S972 and D14S139 at 14q12-q22.1.

Optic disc abnormalities are a group of phenotypically heterogeneous diseases including optic pit, colobomatous optic nerve, morning glory disk anomaly, peripapillary staphyloma, and vacant optic disc^1, 3, 7^, all of which are traditionally considered as distinct ONH anomalies. Currently, these diseases are hypothesized to be resulted from similar developmental defects of the embryonic closure but with various degrees, and therefore, are collectively termed as CODA^1, 9^. In families with inherited CODA, dramatic intra-familial diversities of ONH anomalies were observed ranging from typical pits to large anomalous^1, 3^. Mutations in *PAX6* were also associated with a variety of optic disc defects^5^. These genetic evidences further emphasized the point that same genetic lesion could correlate with distinct diagnoses of ONH anomalies resulting in clinical heterogeneity of CODA. In this sense, classification of CODA is somehow indefinite and confusing^1^, we therefore diagnosed all patients in this family as CODA without further classification. Unlike previously reported large families^1, 3^, appearances of ONH among patients in this family were only moderately varied by size of optic disc and excavation. SD-OCT demonstrated a deep excavation in the center of ONH in all affected eyes for which we had data. In addition, morphology of vasculature changes on ONHs were similar among all patients, showing bilateral missing of central retinal artery and vein with several cilioretinal vessels emerging from the edge of the vacant optic disc. Fundus fluorescein angiography was not available to demonstrate the cilioretinal vessels in the family; however, these vessels are very similar to those described by Parsa and associates^10^ in various vacant optic disc diseases. Remarkable intra-familial phenotypic diversities were also noted in this family, including presence of congenital nystagmus and strabismus, onset age of visual impairment, extension of retinoschisis, and severity in retinal atrophy. A potential explanation for the variation is the existence of a genetic modifier, which regulates the expression of the mutant allele and thus modulates the phenotypic severity^11–13^. In addition, environmental effects may also contribute to the variations.

CODA is often associated with serious retinal detachment or retinoschisis presumably due to the cerebrospinal fluid that enters the vacant disc and travels between retinal layers^14^. However, the occurrences and patterns of retinoshisis were not fully investigated in those previously reported large families due to lack of OCT images. In this family, bilateral retinoschisis were noticed in all patients by fundus examination and FP. Further, SD-OCT revealed peripapillary retinoschisis with various degrees of extension and distinct patterns in all eyes tested. This indicates that retinoschisis in this family is a necessary consequence to optic disc anomalies, but with morphologic variants. Noteworthy, the proband III:8 and patient III:1 were originally misdiagnosed as inherited retinoschisis, which is often juvenile onset, X-linked with mutations in the retinoschisin (*RS1*, MIM: 312700) gene, and involves maculae^15–19^. Macular retinoschisis can also occur in the condition of glaucoma with progressively enlarged excavation^20, 21^. Therefore, differential diagnosis should be carefully made among these diseases by combination of genetic analyses, extensive ophthalmic examinations and inquiries of disease progress.

Mutations in *PAX6* and *PAX2*, two paired box genes that regulate each other, have been implicated in ONH anomalies. *PAX2* is expressed in developing optic stalk, ventral half of the optic cup, and kidney, and its mutations cause papillorenal syndrome^22^. *PAX6* is expressed in developing central nervous system and various ocular tissues^23^, and is a transcription factor required for eye morphogenesis^24^. The only mapped genetic locus linked with CODA is a 13.5 Mbp region on chromosomal 12q^7^. A triplication upstream of the *MMP19* gene is then identified as disease causing for CODA in that family, suggesting the important roles of regulatory factors in the etiology of CODA and other genetic disorders^8^. In our study, we have ruled out coding variants in *PAX6* and *PAX2* in the Chinese family by direct sequencing. Further, genome-wide linkage analysis has mapped the disease locus in the family to the chromosomal region 14q12-q22.1 that does not contain either *PAX6* or *PAX2*, and differs from the previous reported locus on chromosomal 12q. Thus, a new disease-causing gene would be responsible for the CODA phenotypes in this family. The critical interval assigned in this family, spanning about 31 Mbp and containing hundreds of genes, challenges the application of direct sequencing in mutation screening. We therefore employed WES to survey coding variant in nearly all annotated genes. Unfortunately, no putative mutation was identified by WES, which is presumably due to the limitation of the approach on detecting several types of mutation including copy number variation (CNV), large Indels, genome rearrangement and intronic variant. Thus, extensive analyses including whole genome sequencing and CNV screening are further required to call mutation in this family.

## Methods

### Family and clinical examinations

Twelve affected members and 3 presently unaffected siblings from family CL with CODA were included in the present study (Fig. 1). All participants underwent general ophthalmic examinations, including BCVA test, IOP measurement, slit-lamp test, fundus documentation and strabismus examination, and were inquired for systemic disease history or medical record. In addition, VF, electroretinography, SD-OCT, FP, fundus autofluorescence and ophthalmic B-scan were performed on six affected members that were II:3, III:1, III:8, III:11, IV:3 and IV:4. All participants were inquired for detail medical history, especially history of renal diseases. Urinary B-scan and renal function examinations were conducted on three patients (II:1, II:3 and III:1).

Peripheral blood samples were collected from all family members. Genomic DNA was extracted from peripheral blood samples with standard methods. Informed written consent was obtained from each participant for sample collection and molecular analysis. The research was conducted with local ethical approval of the Ethics Committee on Human Research of Zhongshan Ophthalmic Center, according to the Declaration of Helsinki.

### Sanger sequencing of PAX2 and PAX6

Sanger sequencing was used to detect variations in all exons and exon-intron boundaries in genes *PAX2* and *PAX6*. Primers sequences were designed by online program (http://bioinfo.ut.ee/primer3-0.4.0/) (Supplementary Table 4). Polymerase chain reaction (PCR) amplification (35 cycles, 10 seconds at 98 °C, 15 seconds at 60 °C and 2 minutes at 72 °C) was carried out on DNA samples from two affected family members (III:8 and IV:3) with TaKaRa PCR Amplification Kit (Takara Bio Inc., Nojihigashi, Japan). PCR products were then purified and sequenced using an ABI 3730XL Genetic Sequencer in both directions (Applied Biosystems, Foster City, CA, US). Exons with detected variations were next sequenced in all family members to evaluate whether they represent disease-associated mutations.

### Microsatellite markers

A total of 366 polymorphic microsatellite markers (data provided upon request), representing 22 autosomes and X chromosome at approximately 10 cM intervals, were applied for genome-wide linkage screening. Twelve additional microsatellite markers including D14S122, D14S1023, D14S1070, D14S283, D14S972, D14S64, D14S1032, D14S1068, D14S976, D14S978, D14S139 and D14S1057 were further selected for fine mapping on chromosome 14. PCR amplifications (Applied Biosystems) of the microsatellite loci were carried out using fluorescently labeled primers according to a previously described protocol^7^. Genotyping data were collected using GeneMapper 4.1 (Applied Biosystems).

### Linkage analysis and haplotyping

Multipoint linkage analyses were performed using the LINKAGE software package of MERLIN 1.1.2^25^. Parametric linkage analysis of family CL was assumed as an autosomal dominant model with a risk allele frequency of 0.0001 and a penetrance of 99%. Allele frequencies for each marker were assumed to be equal as well as the recombination frequencies in males and females. Haplotypes were constructed using Cyrillic software (version 2.1) and confirmed by inspection.

### Exome sequencing

WES was conducted on two affected individuals (III:8 and IV:3) to reveal the disease-casing mutation. Four μg genomic DNA sample for each individual was fragmented, paired to the ends, ligated with adapters to both ends, and amplified by ligation-mediated PCR. The fragments were hybridized and captured by magnetic beads. Illumina Truseq Exome Enrichment Kit (Illumina Technologies, San Diego, CA, USA) covering over 62 Mbp of the human genome was then employed for enrichment of 20794 genes containing 201121 exons corresponding to the Consensus Coding DNA Sequence Database. Exons, exon-intron sequences, 5′- and 3′-untranslated regions, and non-coding RNAs were all included for mutation screening. High-throughput next generation sequencing was then performed on the Hiseq. 2000 platform (Illumina Technologies). Briefly, all detected variants were initially filtered against 5 SNP databases, including dbSNP137 (http://hgdownload.cse.ucsc.edu/goldenPath/hg19/database/snp137.txt.gz.), HapMap project (ftp://ftp.ncbi.nlm.nih.gov/hapmap), 1000 Genome Project (ftp://ftp.1000genomes.ebi.ac.uk/vol1/ftp), YH database (http://yh.genomics.org.cn/), and Exome Variant Server (http://evs.gs.washington.edu/EVS/). Sanger sequencing was next applied for mutation validation and co-segregation analysis.

## Electronic supplementary material

Supplementary information
