# Supplementary material for: A large family with inherited optic disc anomalies: a correlation between a new genetic locus and complex ocular phenotypes
Source: Sci Rep. 2017 Aug 10;7:7799. doi: 10.1038/s41598-017-07730-7 (PMC5552876; doi:10.1038/s41598-017-07730-7)
Supplement: Supplementary file 1 — Supplementary information [file 41598_2017_7730_MOESM1_ESM.doc]

**A Large Family with Inherited Optic Disc Anomalies:  a Correlation between a New Genetic Locus and complex ocular phenotypes**

Decai Wang, Xinyuan Pan, Jiangdong Ji, Shun Gu, Xiantao Sun, Chao Jiang, Weiyi Xia, Zhihua Qiu, Xiaoli Kang, Sijia Ding, Qinghuai Liu, Xue Chen, Fang Lu, Chen Zhao

**Supplementary information file**

Supplementary information file includes one Figure (**Figure S1**) and three Tables (**Table S1**-**S4**).

**Legends to Figure S1**

**Figure S1. Visual field test results for patients in family CL.**

Visual field test results of patient II:2 (A), III:1 (B), III:9 (C), III:14 (D), and IV:3 (E).


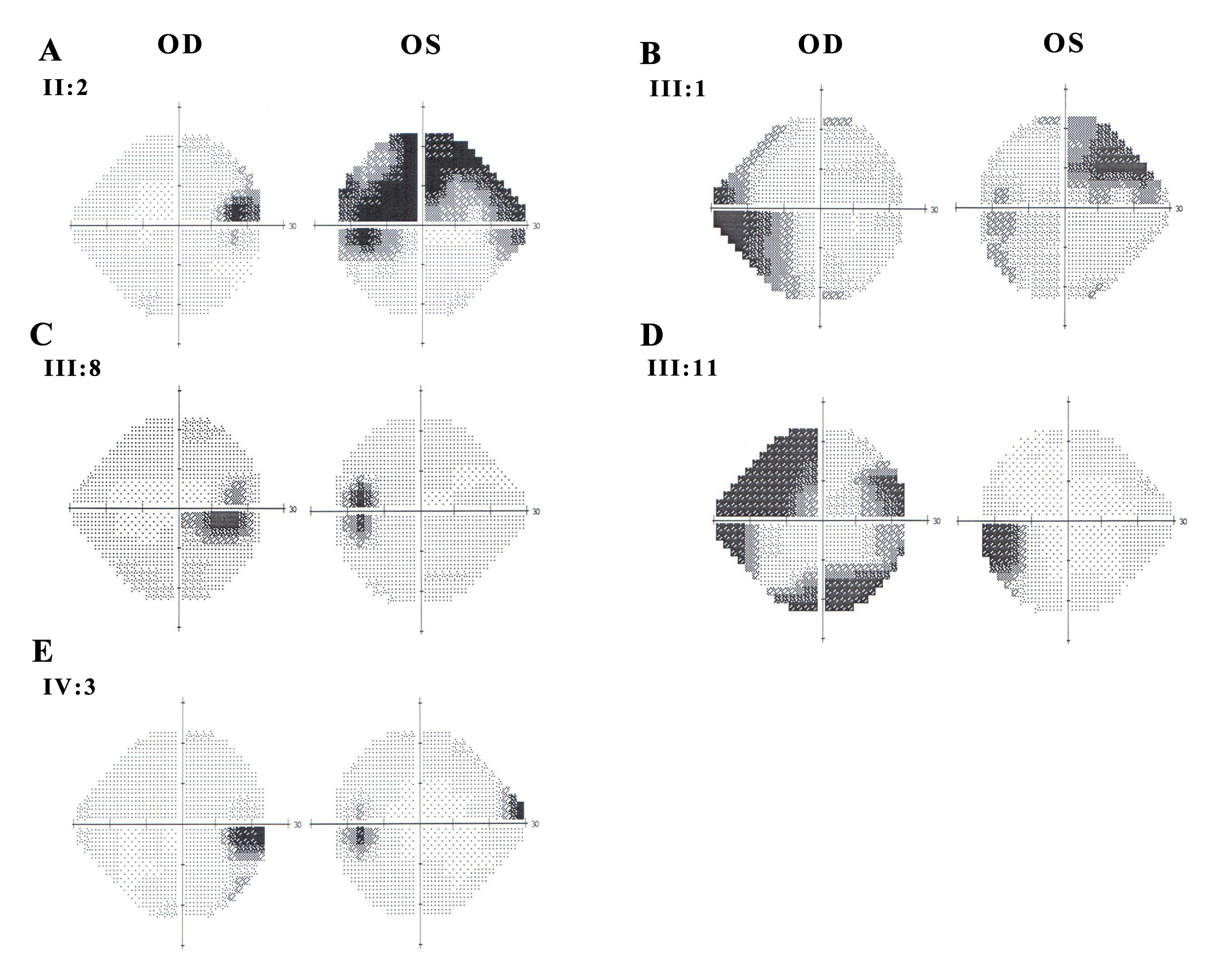


| **Supplementary Table 1. Microsatellite markers with multiple LOD>0.** | | | |
| --- | --- | --- | --- |
| **Chr** | **Markers** | **Genetic Marshfield**  **(cM)** | **LOD Scores**  **(penetrance 99%)** |
| 3 | D3S1265 | 222.83 | 0.229 |
| 9 | bt. D9S161 & D9S1817 | 55.575 | 0.992 |
| 10 | D10S185 | 116.34 | 0.527 |
| 10 | … | 120.305 | 0.671 |
| 10 | D10S192 | 124.27 | 0.742 |
| 10 | bt. D10S192 & D10S1693 | 130.83 | 0.444 |
| 10 | bt. D10S1693 & D10S587 | 142.48 | 0.228 |
| 14 | bt. D14S261 & D14S990 | 10.53 | 1.587 |
| 14 | D14S990 | 14.6 | 1.432 |
| 14 | … | 21.305 | 3.247 |
| 14 | D14S275 | 28.01 | 3.726 |
| 14 | … | 34.06 | 3.742 |
| 14 | D14S70 | 40.11 | 3.804 |
| 14 | … | 43.81 | 3.813 |
| 14 | D14S288 | 47.51 | 3.84 |
| 14 | bt. D14S288 & D14S276 | 51.935 | 3.235 |
| 18 | D18S452-D18S464 | 24.935 | 0.174 |
| **Abbreviations:** Chr, chromosome; cM, centi Morgan; bt., between; …, refers a location between up and below marekers. | | | |

| **Supplementary Table 2. LOD scores of microsatellite markers on Chr12.** | | | | | |
| --- | --- | --- | --- | --- | --- |
| **STR Marker** | **Genetic Marshfield (cM)** | **LOD (penetrance 80%)** | **LOD (penetrance 90%)** | **LOD (penetrance 99%)** | **LOD (penetrance 100%)** |
| D12S352 | 0 | -12.448 | -13.466 | -15.766 | -36.091 |
| D12S1725 | 9.52 | -6.539 | -7.043 | -7.921 | -8.16 |
| D12S336 | 19.68 | -5.972 | -6.579 | -8.468 | -22.142 |
| D12S310 | 36.06 | -5.98 | -6.652 | -8.695 | -28.578 |
| D12S1617 | 44.03 | -4.52 | -5.109 | -7.077 | -28.871 |
| D12S345 | 53.09 | -8.933 | -9.517 | -10.798 | -20.832 |
| D12S85 | 61.34 | -9.979 | -10.49 | -11.66 | -14.756 |
| bt D12S85  & D12S83 | 68.255 | -4.658 | -5.035 | -5.772 | -5.992 |
| D12S83 | 75.17 | -4.223 | -4.868 | -6.891 | -16.835 |
| D12S326 | 86.4 | -3.411 | -4.04 | -6.045 | -16.224 |
| D12S351 | 95.56 | -7.003 | -8.197 | -10.253 | -31.957 |
| D12S346 | 104.65 | -4.189 | -4.379 | -4.642 | -4.684 |
| D12S78 | 111.87 | -5.295 | -5.476 | -5.67 | -5.696 |
| D12S1583 | 119.55 | -2.189 | -2.228 | -2.287 | -2.296 |
| D12S1718 | 128.05 | -6.213 | -6.799 | -8.201 | -14.035 |
| D12S304 | 144.83 | -8.007 | -8.684 | -10.731 | -15.058 |
| D12S1659 | 155.94 | -9.534 | -9.932 | -11.001 | -15.017 |
| D12S1723 | 164.63 | -3.633 | -4.082 | -5.241 | -8.537 |

| **Supplementary Table 3. Variants heterozygously shared by both screened cases and located within the interval.** | | | | | |
| --- | --- | --- | --- | --- | --- |
| **Chr** | **Position** | **Gene** | **Ref Allele** | **Alt Allele** | **Genetic Region** |
| 4 | 24620867 | *RNF31* | G | T | exonic |
| 14 | 24701610 | *GMPR2* | C | A | upstream |
| 14 | 25518919 | *STXBP6* | C | A | 5’-UTR |
| 14 | 39784042 | *CTAGE5* | A | G | intronic |
| 14 | 39784044 | *CTAGE5* | A | G | intronic |
| 14 | 50735957 | *L2HGDH* | C | T | exonic |
| 14 | 51060743 | *ATL1* | A | G | intronic |

| **Supplementary Table 4. Primers of *PAX2* & *PAX6* genes.** | | | | | |
| --- | --- | --- | --- | --- | --- |
| **Genes** | **Exons** | **Length**  **(bp)** | **Annealing**  **temperatures (°C)** | **Forward primers (5'→3')** | **Reward primers (5'→3')** |
| *PAX2* | 1 | 829 | 62 | AGTCTCCGGCCGAGTCTTCT | GCCTCCAAGATGGGACCT |
|  | 2 | 459 | 58 | AGTCTTCAGCCCAGCGTCT | GCTGGACTTTTAGCCACGTC |
|  | 3 | 498 | 60 | AAGTCAGCTCAGCCACACTG | TCACAGGTTCCCTTTCTCTGA |
|  | 4 | 391 | 60 | GAAATCGCTGAGGAACTTGG | GTGGGAGGCAGAGAACAGTC |
|  | 5 | 367 | 59 | GGCTCCTCATCCCTCCTTAT | GGACCTGGGCTTTGCTACTA |
|  | 6 | 493 | 60 | ATTGCAGCTCAGAACCCTTG | CCCGATCTGCTCTTTGTTTT |
|  | 7 | 490 | 61 | TTCTCTTCCTTGGGCTTCCT | CACATACAATGCTGGCTATGC |
|  | 8 | 477 | 61 | TGCCCCACCATCTCTTTCTA | CCTAGCATCAGCCTTTCCAG |
|  | 9 | 250 | 60 | CAGTACCCTGGTGTGAGTAGAGG | CTACAGACCATTCAGCAGCTC |
|  | 10 | 698 | 60 | CCCAATACAAACCCTTCGTG | AGTTGCCCCGGAGAATAGTT |
|  | 11.1 | 921 | 60 | AACTATTCTCCGGGGCAACT | CCAACCTTGGAAAGACCTGA |
|  | 11.2 | 978 | 61 | AAATTCTTTTCCCCCAGTGC | CCATGTTCGTCATTTTGCAT |
|  | 11.3 | 991 | 60 | TACACGCCCATTAAAGCACA | GGATCATTGAAGGGGCTTTT |
| *PAX6* | 1 | 492 | 59 | CAAGGAAGGCCAAAGCAG | TCGCTTCCATCTTTGTATGC |
|  | 2 | 467 | 60 | ACACACTTGAGCCATCACCA | CTCCTGCGTGGAAACTTCTC |
|  | 3 | 400 | 60 | GTGGGTGTAATGCTGGGACT | CCCAATCTGTTTCCCCTACA |
|  | 4 | 488 | 62 | CAGCTGCCCGAGGATTAACT | GCCTCCTTCTCGCTCAACTA |
|  | 5 | 327 | 57 | CTCCCTCATCTTCCTCTTCC | GGGGTCCATAATTAGCATCG |
|  | 6.7 | 509 | 60 | GGGCTACAAATGTAATTTTAAGA | AGAGAGGGTGGGAGGAGGTA |
|  | 8 | 300 | 58 | GAGCTGAGATGGGTGACTG | GAGAGTAGGGGACAGGCAAA |
|  | 9 | 291 | 61 | AGACTACACCAGGCCCCTTT | TGAAGATGTGGCATTTACTTTGA |
|  | 10 | 243 | 62 | GGAACCAGTTTGATGCACAG | ACTCTGTACAAGCACCTCTGTCTC |
|  | 11 | 300 | 62 | GGGCTCGACGTAGACACAGT | GGAAACTGAGGGCAAGAGAA |
|  | 12 | 361 | 60 | GCACCAGTGTCTACCAACCA | AAAGCTCTCAAGGGTGCAGA |
|  | 13 | 281 | 61 | GCTGTGGCTGTGTGATGTGT | AGGAGATTCTGTTTGGGTA |
|  | 14.1 | 786 | 60 | TCCATGTCTGTTTCTCAAAGG | CGAAGACACACTCTACCTTTTAGC |
|  | 14.2 | 978 | 60 | AAACCTGGAACAACATGCACT | AAGGGAGAGGGCCTATTTGA |
|  | 14.3 | 993 | 60 | ATCTGGGCAGATTTCCATTG | CGGCAATTGTACCAACAGTG |
|  | 14.4 | 961 | 61 | ACTGCACAGCAGCACATTTC | TGGCAGTGAGCTGTAGCAAG |
|  | 14.5 | 844 | 60 | CCCTTAAATGGTGAACAACTGG | CAATGCCAAACGTAATATCTG |
|  | 14.6 | 849 | 59 | TCTCATGTTAAAGAAAGGCA | AATGTTGTGCGGATACTCCA |
|  | 14.7 | 871 | 60 | AGACATGGTAGCCAGGGAAG | CATCCATTGGGAGAAACTGAA |
| **Abbreviation:** bp, base pair | | | | | |
